# Environmental pressures and pesticide exposure associated with an increase in the share of plant-based foods in the diet

**DOI:** 10.1038/s41598-023-46032-z

**Published:** 2023-11-07

**Authors:** Emmanuelle Kesse-Guyot, Benjamin Allès, Joséphine Brunin, Brigitte Langevin, Hélène Fouillet, Alison Dussiot, Florine Berthy, Anouk Reuzé, Elie Perraud, Pauline Rebouillat, Mathilde Touvier, Serge Hercberg, François Mariotti, Denis Lairon, Philippe Pointereau, Julia Baudry

**Affiliations:** 1 Nutritional Epidemiology Research Team (EREN), Epidemiology and Statistics Research Center (CRESS), Sorbonne Paris Nord University and University of Paris, Inserm, INRAE, Cnam, 74 Rue Marcel Cachin, 93017 Bobigny, France; 2 ADEME, Agence de l’Environnement et de la Maîtrise de l’Energie, 49004 Angers, France; 3 UMR PNCA, AgroParisTech, INRAE, Paris-Saclay University, 75005 Paris, France; 4 Solagro, 75, Voie TOEC, CS 27608, 31076 Toulouse Cedex 3, France; 5 Département de Santé Publique, Hôpital Avicenne, 93017 Bobigny, France; 6 INSERM, INRAE, C2VN, Aix Marseille Université, 13005 Marseille, France

**Keywords:** Environmental sciences, Planetary science, Risk factors

## Abstract

Diets rich in plant-based foods are encouraged for human health and to preserve resources and the environment but the nutritional quality and safety of such diets is debated. This study aimed to model nutritionally adequate diets with increasing plant food content and to characterise the derived diets using a multicriteria approach including, nutrients intake, environmental pressures and exposure to pesticides. Using data of the NutriNet-Santé cohort (N = 29,413), we implemented stepwise optimization models to identified maximum plant-food content under nutritional constraints. Environmental indicators at the production level were derived from the DIALECTE database, and exposure to pesticide residues from plant food consumption was estimated using a contamination database. Plant-based foods contributed to 64.3% (SD = 10.6%) of energy intake in observed diets and may reach up to 95% in modelled diets without jeopardizing nutritional status. Compared to the observed situation, an increase in plant-based foods in the diets led to increases in soy-based products (+ 480%), dried fruits (+ 370%), legumes (+ 317%), whole grains (+ 251%), oils (+ 144%) and vegetables (+ 93%). Animal products decreased progressively until total eviction, except for beef (− 98%). Dietary quality (estimated using the Diet Quality Index Based on the Probability of Adequate Nutrient Intake) was improved (up to 17%) as well as GHGe (up to − 65%), energy demand (up to − 48%), and land occupation (− 56%) for production. Exposures to pesticides from plant-based foods were increased by 100% conventional production and to a much lesser extent by 100% organic production. This study shows that shifting to nutritionally-adequate plant-based diets requires an in-depth rearrangement of food groups’ consumption but allows a drastic reduction environmental impact. Increase exposure to pesticide residues and related risks can be mitigated by consuming foods produced with low pesticide input.

## Introduction

Modern western diets, rich in animal products and salt, saturated fat, and sugar, are not sustainable^1^. Responsible for many chronic diseases, western diets also have harmful consequences on natural resources and strongly contribute to climate change^2,3^. Since 1950s, population’s growth, modernization and urbanization have led to an intensification of agriculture. In addition, increased wealth is associated with increased animal-based foods demand^4^. However, production of animal food for humans is very inefficient in terms of energy, especially in intensive production settings^5^, since a loss of energy occurs throughout the trophic chain.

Indeed, the scientific literature robustly documents that food systems, particularly intensive and industrialized ones, are responsible for major environmental degradation, such as deforestation, water use and greenhouse gas emissions (GHGe)^6,7^. Additionally, the production of meat, fish, eggs and dairy products uses ~ 83% of agricultural land globally and contributes 56–58% of the emissions generated by food production, while providing 37% of the protein supply^8^. Meanwhile some extensive grazing systems in Europe contribute to High Nature Value farmland^9^ and overall, changes in farming practices may help in mitigate harmful impacts^10^.

This explains the drastically lower environmental footprint of plant-based diets and even more for vegetarian or vegan diets^11–15^. Indeed, observational or modelling studies show that the reduction of animal products in diets is associated with lower environmental pressures, considering mostly indicators related to climate change or land use^16^. For example, we have recently shown that a moderate reduction of GHGe was associated with a gradual increase of fruits, vegetables and soy-based products in the diet and conversely a decrease of animal products^17^. However, existing optimization studies do not consider potential difference in environmental pressures according to farming practices.

Plant-based diets have been consistently associated with long-term health, i.e. lower risk of chronic diseases^2,18^. However, plant-based foods include both healthy and unhealthy foods, such as ultra-processed foods and/or sweetened beverages, desserts, or salty or sweet snacks, so it is important to clarify which healthy and sustainable plant-based products should be substituted for animal-based products^19,20^. An issue frequently raised, beyond social norms associated with animal-food consumption and taste, is related to the nutrients constituting the animal, versus plant-based, protein package, which may compromise nutritional status for protein, zinc, iron and vitamin B12^21^. However, it has been shown that in Western countries protein undernutrition is rare (except for the elderly or frail) insofar as if total protein requirements are covered, amino acid intakes are not limiting^22^. Some authors have also emphasized that the amount and quality of plant-protein is often underestimated or misunderstood^23^. In addition, we have recently shown in an optimization study that it is possible to eliminate meat from the diet and that dietary changes can meet the requirements for iron, zinc, and vitamin A. In that study, other nutrients supplied mainly by meat, such as vitamins B6 and B12, proteins and essential amino acids, were never limiting^24^.

Besides, we have shown that a reduction in the consumption of animal products (meat and dairy products) leads to potentially insufficient intakes of iron and zinc based on official recommendations^17^, but the latter may be overestimated^25^.

Some authors have qualified the increase in demand for protein-rich foods (related to population growth and socio-economic development), high biological value of animal proteins, and the low environmental pressures of plant protein as a “*protein trade-off*” (“human versus ecosystem health”)^26^. In addition, another issue pertained to the potential elevated chronic exposure to pesticide residues that are strongly associated with plant-food consumption at the individual level even if food maximal level of residues are mostly respected^27^. We previously showed that potential exposure to pesticides residues may be drastically increased for people with highly plant-based diet^28^.

The primary objective of the present study was to identify optimized diets gradually higher in plant-based foods (expressed as energy and noted %PE) but fully adequate in all nutrient intakes (including those conveyed by animal foods, i.e. under nutritional and acceptability constraints), while considering the beef/milk coproduct link. A second objective was to study the externalities of these diets by describing the optimized diets in terms of environmental pressures and to evaluate pesticide residue exposure associated with diet increased in plant-food.

## Methods

### Population

This analysis is based on a sample of adults involved in the ongoing web-based prospective NutriNet-Santé cohort aiming to investigate relationships between nutrition and health^29^. Participants are recruited on a voluntary basis from the general French population. This study is conducted in accordance with the Declaration of Helsinki, and all procedures were approved by the Institutional Review Board of the French Institute for Health and Medical Research (IRB Inserm 0000388FWA00005831) and the National Commission on Informatics and Liberty (Commission Nationale de l’Informatique et des Libertés, CNIL 908,450 and 909,216). Electronic informed consent was obtained from all participants. The NutriNet-Santé study is registered in ClinicalTrials.gov (NCT03335644).

### Dietary data assessment

**Figure 1:**
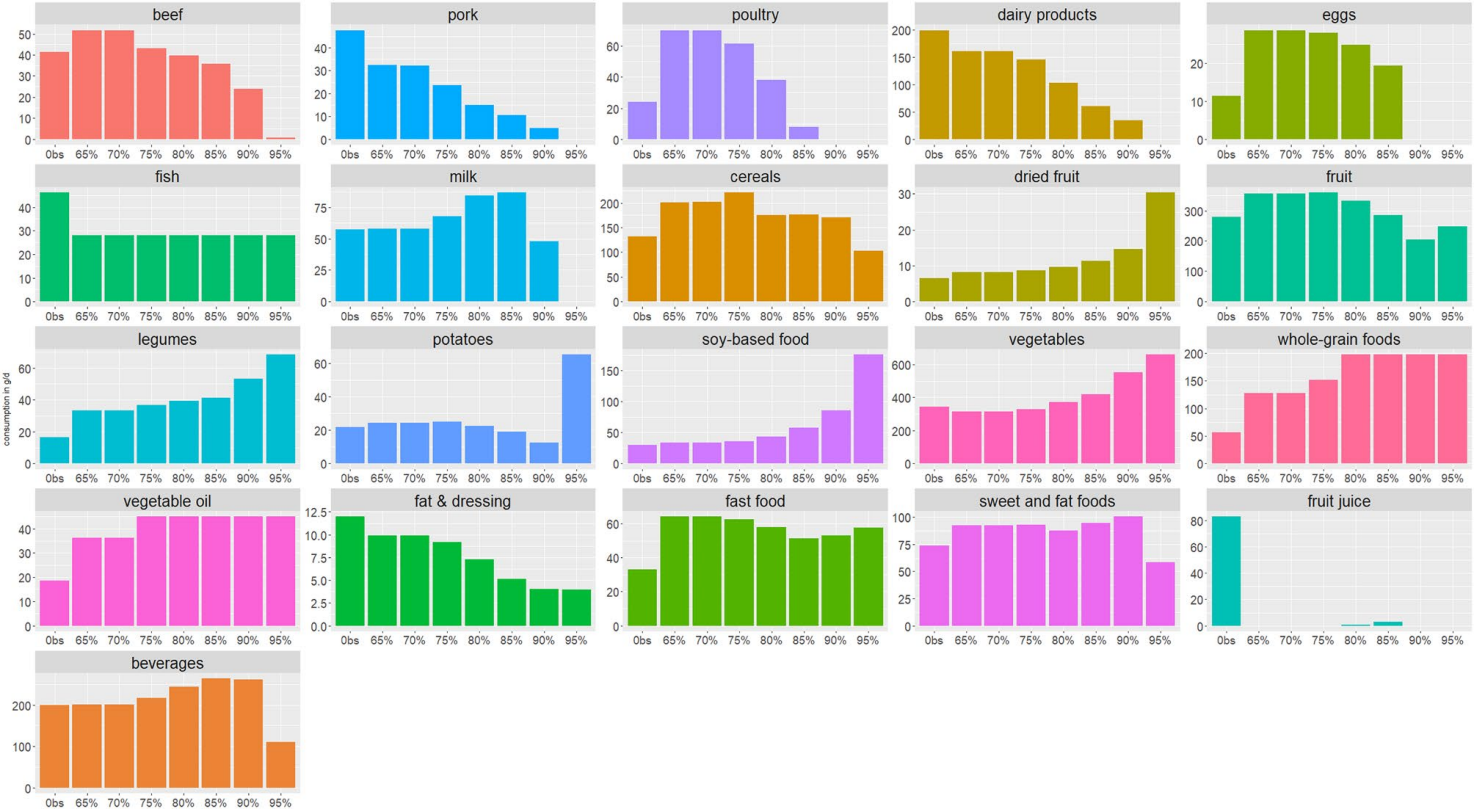
Composition (g/d) of the observed and optimized scenarios modelling modelled diets with gradual increase in the proportion of energy intake from plant-based foods^1,2^. Abbreviations: Obs, observed diet. ^1^Food group consumption (g/d) in the observed diets and in the modelled diets being nutritionally, culturally and environmentally optimized so as to ensure gradual increase in the proportion of energy intake from plant-based foods. The basal scenario (65%) correspond to the modelled diet when the proportion of energy intake from plant-based foods is set at the observed value of proportion of energy intake from plant-based foods under nutritional, fish consumption limitation and coproducts constraints. Next scenarios increase plant-based foods energy from 65 up to 95%. ^2^Vegetables include all vegetables and soups, fruit include fresh fruit, fruit in syrup and compote, dried fruit and seeds, fish include seafood, dairy product include yogurts, fresh cheese and cheese, potatoes include other tubers, cereals include breakfast cereal low in sugar, bread semolina, rice and pasta, sweet and fat foods include croissants, pastries, chocolate, biscuits, milky dessert, ice cream, honey and marmalade, cakes, chips, salted oilseeds, salted biscuits, beverages include fruit nectar, syrup, soda (with or without sugar), plant-based beverages (except soy-based), milk consumed with tea/coffee, fast-food include sandwich, prepared foods such as pizza, hamburger, ravioli, panini, salted pancake, etc., soy-based food include tofu, soy meat substitute and vegetable patties, soy yogurt, soy milk, and fats include fresh cream and butter.

The dietary data were collected using a self-administered validated semi-quantitative food frequency questionnaire (FFQ), administered from June to December 2014, extensively described elsewhere^30^. For each of the 264 food and beverage items, the questionnaire has been augmented by a five-point ordinal scale to evaluate the mode of production, i.e. organic (under official label) or conventional^31^. Thus, participants were asked to choose among the following answer modalities: “never”, “rarely”, “half-of-time”, “often” or “always” in response to the question ‘How often was the product of organic origin?’. Organic food consumption was estimated by allocating the respective weights: 0, 25, 50, 75 and 100% to the modalities. Consumption reports are for foods as consumed and edible part coefficients have been applied. For clarity, food and beverage items were grouped into food groups specifically defined for this optimization modelling (see footnotes to Fig. 1). Nutritional composition of each item was determined by combining the published NutriNet-Santé food composition table (> 3000 items)^32^ with the 264 FFQ-items as the weighted mean of the nutritional content of all corresponding foods. For each food, energy intake from plant or animal source was calculated. Energy intake from plant or animal sources was calculated based on validated recipes developed by dieticians, taking into account the nature of the ingredients.. Weights were the frequencies of consumption in the NutriNet-Santé population. Individual nutrient intakes were calculated.

### Sociodemographic and lifestyle characteristics

Age, education (< high school diploma, high school diploma, and post-secondary graduate), smoking status (former, current, or never-smoker), and physical activity assessed using the International Physical Activity questionnaire^33^ were collected using pre-validated questionnaires updated each year^34,35^. For this study, we used the data closest to the FFQ completion date.

### Dietary indicators

The nutritional quality of the optimized diets was assessed using three dietary indexes.

The nutrient-based PANDiet (Diet Quality Index Based on the Probability of Adequate Nutrient Intake) contains two subscales reflecting *adequacy* and *moderation*^36^. For each nutrient, the ‘probability of adequacy’, i.e. intake above minimum values (*adequacy* score) or below maximum values (*moderation* score) is calculated on the basis of nutrient reference values. The final score is the average of the two sub-scores. The adequacy sub-score is the average of the probabilities of adequacy for 28 nutrients and the moderation sub-score includes 6 nutrients and 12 penalty values referring to the probabilities of exceeding upper limits of intakes. The PANDiet ranges from 0 to 100 points, with a higher score reflecting better adherence to French nutritional recommendations and adequate nutrient intake. The calculation to estimate the adequacy of the usual intake for a given nutrient is as follows:

$$ Prob\left( {\frac{y - r}{{ SDr}}} \right) $$

where Prob: is the *probnorm* function of SAS®, y: daily mean intake, r: the reference value, SDr: the interindividual variability.

The second score was based on food group consumption and has been recently developed to assess the quality of plant and animal foods^37^. Each component (healthy/unhealthy plant-based/animal food consumption) ranged between 0 and 5 points for a total maximum score of 85. Components and scoring are presented on Supplemental Fig. 1.

Third, the sPNNS-GS2 is a validated score, ranging from − ∞ to 14.25, reflecting adherence to the 2017 French food-based dietary guidelines proposed by the High Council of Public Health^38,39^. It is composed of 12 weighted components for moderation or adequation and penalty for energy intake was applied. Components and scoring are presented in Supplemental Fig. 2.

### Environmental pressure indicators

Food-related environmental indicators were computed using upstream life cycle analysis (LCA) from the DIALECTE database developed by Solagro^40^. This database has the particularity of covering conventional and organic farms. GHGe (kg of CO_2_ equivalents (CO_2_eq)), cumulative energy demand (MJ), and land occupation (m^2^) were computed at the farm perimeter excluding downstream steps such as conditioning, transport, processing, storage or recycling stages. Details, data and computation have been broadly described elsewhere^41^.

Data of 92 raw agricultural products, economic allocation (accounting for coproducts), as well as cooking and edibility coefficients were used to estimate environmental pressures related to the production of the 264 food items. Pesticide residue exposure.

A food contamination database was developed from the data provided by the CVUA in Stuttgart. It consists of 6 billion data points on pesticide residue levels collected in Europe during the period 2012–2015 for foods of plant origin, both organic and conventional (the database does not cover foods of animal origin, which are known to be much less contaminated by pesticide residues than foods of plant origin). The data collection and computation have been extensively described elsewhere^30^. The plant-based food of the FFQ were decomposed into 442 ingredients for which the mean of contamination for a list of compounds was computed. Pesticide residues included active substances, such as organophosphates, pyrethroids, others and active substances allowed in organic farming such as natural pyrethrins and spinosad. A synthetic indicator was calculated as the sum of exposure inverse weighted on the Acceptable Daily Intake (ADI).

### Coproduct factors for ruminant products

As previously published^17^, we considered a coproduct factor between milk and beef. Indeed, increase in plant protein is associated with a decrease in beef consumption. However, in particular to meet calcium requirement, milk consumption is not suppressed implying that cattle breeding persists.

In 2010 in France, 25 million tons of milk and 1.52 million tons of beef (expressed in carcass weight)^42^ were produced, of which 41% was from dairy herd, i.e., 0.62 million tons of beef^43^. Considering a meat to carcass weight ratio of 68%^44^, and further yields of 90% during distribution (due to 10% distribution losses) and 68% during consumption (due to 32% losses by cooking, bones and wastes)^44^ and that 1L of milk corresponded to 10 g of meat when applying the equation:

$$ \begin{aligned} {25}\;{\text{million}}\;{\text{tons}}\;{\text{of}}\;{\text{Milk }}\left( L \right)\, = & 1.52\;{\text{ million}}\;{\text{tons}}\;{\text{of}}\;{\text{beef}} \times 41\% { } \times 68\%_{carcass\; yield} \\ & \times 90\%_{distribution\;yield} \times 68\%_{preparation\;yield} \\ \end{aligned} $$

### Weighting of nutritional reference

The nutritional reference values are established separately for men and women since they have significantly different physiological requirements^45^. In addition, a subsequent distinction is made between women with high vs low iron requirements. It is estimated that about 20% of menstruating women have high iron requirements^45^. In this study, to improve clarity, we defined an average individual constituted of 50% men and 50% women, reflecting the French distribution. In addition, for women, we considered 50% postmenopausal women and 50% non-menopausal women with low and high iron requirement respectively. The assignment of high iron requirements to all menstruating women allows to mimic the strictest situation. Reference values for each nutrient were defined as the weighted mean and are presented in Table 1.

**Table 1:** Nutritional constraints used in the optimization models.

	Unit	Men		Women		Average individual^a^	
		Lower reference	Upper reference	Lower reference	Upper reference	Lower reference	Upper reference
Energy intake	Kcal/d	ER−8%	ER + 8%	ER−8%	ER + 8%	ER−8%	ER + 8%
Protein	kg of BW/d	0.83	2.3	0.83	2.3	0.83	2.3
Vitamin A	µg/d	750	3000	650	3000	700	3000
Vitamin B1	µg /1000 kcal/d	0.3	–	0.3	–	0.3	–
Vitamin B2	mg /1000 kcal/d	0.55	–	0.55	–	0.55	–
Vitamin B3	µg /1000 kcal/d	5.44	900	5.44	900	5.44	900
Vitamin B5	mg/d	P5	–	P5	–	Weighted P5	–
Vitamin B6	mg/d	1.7	25	1.6	25	1.65	25
Vitamin B9	µg/d	330	–	330	–	330	–
Vitamin B12	µg/d	4	–	4	–	4	–
Vitamin C	mg/d	110	–	110	–	110	–
Vitamin E	g/d	P5	–	P5	–	Weighted P5	–
Vitamin K	µg/d	P5	–	P5	–	Weighted P5	–
Calcium	mg/d	950	2500	950	2500	950	2500
Copper	mg/d	P5	5	P5	5	Weighted P5	
Bioavailable Iron	mg/d	1.76	–	2.56 / 1.76^b^	–	1.92	–
Iodine	µg/f	150	600	150	600	150	600
Magnesium	mg/d	P5	–	P5	–	Weighted P5	–
Manganese	mg/d	P5	–	P5	–	Weighted P5	–
Phosphorus	mg/d	550	–	550	–	550	–
Potassium	mg/d	3500	–	3500	–	3500	–
Selenium	µg/d	70	300	70	300	70	300
Sodium	mg/d	1500	2300	1500 mg	2300	1500	2300
Bioavailable zinc	mg/d	(0.642 + 0.038 kg of body weight)		(0.642 + 0.038 kg of body weight)		3.3^3^	
Saturated fatty acids	% EI/d	–	12	–	12	–	12
Linoleic acid	% EI/d	4	–	4	–	4	–
Alpha-linolenic acid	% EI/d	1	–	1	–	1	–
Linoleic acid / alpha-linolenic acid	–	–	5	–	5	–	5
Eicosapentaenoic acid + docosahexaenoic acid	g/d	0.5	–	0.5	–	0.5	–
Sugar without lactose	g/d	–	100	–	100	–	100
Fibre	g/d	30	–	30	–	30	–

ER, energy requirement.

^a^The average individual was the weighted mean as follows: 50% men, 25% women M^-^, 25% M^+^.

^b^High and low iron requirements.

For mean, 5th and 95th percentiles (see below) values of observed food item intakes, we calculated weighted averages after calculation of individual weights so that the proportions defined above were respected.

### Modelling the increase of the contribution of plant food to energy intake

Using non-linear optimization modelling, we identified optimized consumptions of 264 different food items, as well as their respective proportions in organic. We obtained optimized diets with minimal diet departure from the initial (observed) diets, while maintaining a set of constraints including nutritional (adequate nutrient intakes), acceptability, and coproduction constraints. An additional constraint was imposed, which was the gradual increase of the percentage of energy obtained from plant-based foods until the maximal value identified in a preliminary step. Optimized nutritionally adequate diets were developed from initial conditions based on observed food consumptions and nutritional composition of food items^46,47^.

The list of fixed constraints was as follows:

- Nutritional constraints on daily energy intake and a set of nutrients were defined according to the upper and/or lower reference values. Lower bounds were defined as recommended dietary allowance (population reference intake), adequate intake, or lower bound of reference range for the intake in the French population of ANSES^45^ based on the 2021 EFSA opinion^48^. For adequate intake based on observed mean intake, the lower limit was set at the weighted 5th percentile value. Upper bounds were defined as the maximum tolerable intakes for vitamins and minerals when available, or the upper limit of the reference intake range otherwise.
- For zinc and iron, bioavailability was considered using the published formula^49,50^. Further details are presented in Supplemental Material.
- Acceptability constraints were defined at the food group level, with upper bounds set at the weighted 95th percentiles values.
- To comply with some contaminant constraints, such as heavy metals, we added a constraints as regarding total fish consumption (≤ 2 portions / week)^39^.
- Coproduction constraint limited the consumption of milk to a proportion of that of beef, using the factor between milk and beef defined as reported above.

The modelling process was conducted in two steps:

In the first model, we searched to identify the maximal contribution of plant-based foods to diet energy (%PE) satisfying the all the fixed constraints, and the objective function was hence defined as the equation:

$$ {\text{Max }}\% {\text{PE}} = \mathop \sum \limits_{i}^{264} \left[ {\frac{{Kcal - Plant_{i} \times Opt_{i} }}{{Kcal_{i} \times Opt_{i} }}} \right] $$

where i is the food item, Kcal-Plant_i_ and Kcal_i_ denote plant and total energy value in the food item (i), respectively and Opt_i_ denote the daily consumption of the food item (i) in the optimized model.

Next, in the main stepwise models, for identifying a culturally acceptable dietary trajectory towards that maximal plant-derived energetic content, plant-energy was included as a gradual additional constraint (in addition to the fixed constraints) following this equation:

$$ \% {\text{PE }} \ge \lambda \% \Leftrightarrow  \mathop \sum \limits_{i}^{264} \left[ {\frac{{Kcal - Plant_{i} \times Opt_{i} }}{{Kcal_{i} \times Opt_{i} }}} \right] \times 100\, \ge \,\lambda \% $$

where i is the food item, Kcal-Plant_i_ and Kcal_i_ denotes plant and total energy value for the food item (i), respectively and Opt_i_ denotes the daily consumption of the food item (i) in the optimized model. λ ranges from the observed value (65%) to the maximum identified in the preliminary step by 5% increment.

The objective function was to minimize at each step the total departure (TD) from the previous modelled diet, as the equation:

$$ {\text{Min}}\;{\text{TD}} = \mathop \sum \limits_{i}^{264} \left[ {\frac{{Opt_{i\left[\kern-0.15em\left[ n \right]\kern-0.15em\right]} - Opt_{{i\left[\kern-0.15em\left[ {n - 1} \right]\kern-0.15em\right]}} }}{{SD_{i} }}} \right]^{2} $$

where $Opt_{i\left[\kern-0.15em\left[ n \right]\kern-0.15em\right]}$ and $Opt_{{i\left[\kern-0.15em\left[ {n - 1} \right]\kern-0.15em\right]}}$ denote the daily consumption of food item (i) in the n and n−1 optimized models, respectively and SD_i_ was the standard deviation of the daily consumption of food item (i) over the whole population in the initial (observed) condition.

Diet optimization was performed using the procedure SAS/OR ® *optmodel* (version 9.4; SAS Institute, Inc.) using a non-linear optimization algorithm with multi-start option to warrant that identified solutions were not only local optima^47^.

For each model, we conducted an analysis of the standardized dual values to identify the most so-called active constraints of the model, i.e. constraints limiting the objective gain, i.e. minimizing diet departure while complying with all the constraints, compared to the inactive variables that do not drive the model.

This allowed the identification of limiting nutrients This analysis was performed using an approach described in a previous work^51^, by calculating the standardized dual values corresponding to the potential gain in objective in the case of a 100% relaxation of the limiting bound of the constraint^52^”.

### Statistical analysis

For the baseline situation of the present study, we considered participants of the NutriNet-Santé study who had completed the Org-FFQ between June and December 2014 (N = 37,685), with no missing covariates (N = 37,305), not detected as under- or over-energy reporter (N = 35,196), living in mainland France (N = 34,453), and with available data regarding the place of purchase for the computation of the dietary monetary cost as published elsewhere^53^, leading to a final sample of 29,413 participants (Supplemental Fig. 3). The sociodemographic and lifestyle characteristics of the three initial populations (men, premenopausal and menopausal women) and of the average individual were estimated as mean (SD) or percentage.

The optimized diets identified were described for the average individual by the following indicators:

1. Dietary consumption by food groups,
2. Relevant nutrients intakes, as regards plant to animal food rebalancing,
3. Environmental pressures (GHGe, cumulative energy demand and land occupation),
4. Exposure to pesticide residues for two scenarios (100% conventional and 100% organic). To do that, we applied the method as recommended by WHO^54^. For each active substance, the estimated daily intake (EDI) (in µg/kg body weight/d) was calculated under a lower-bound scenario, using the reference method described by Nougadère et al.^55^, combining food consumption, contamination, farming practices and body weight after applying edible coefficients for cooking and peeling. A synthetic indicator of exposure was calculated as the average exposure to each molecule.

Secondary analyses were conducted. First, all the procedures were repeated across 3 subgroups (based on tertile value of the distribution of protein from plant-foods to total) with different values of %PE at baseline: 50%, 65% and 80%. Second, all the procedures were repeated by modelling the increase in the ratio of plant protein instead of the ratio of energy from plants.

All statistical analyses were performed using SAS® (version 9.4; SAS Institute, Inc., Cary, NC, USA) and figures were drawn using R version 3.6. The non-linear optimization problem was performed using the NLP solver of the OPTMODEL procedure of SAS software version 9.4 (SAS Institute Inc., Cary, NC, USA).

## Results

The characteristics of the reference population are presented in Supplemental Table 1. This population initially included 29,413 participants (75% women), with a mean age of 54.5 y. The characteristics of the average individual are also presented. In the observed diet, the proportion of energy intake from plant-based foods was on average 65%.

The first model, aiming at identifying the maximum part of plant-based foods (expressed as a percentage of diet energy) in the diet under nutritional (nutrient requirements by taking iron and zinc bioavailability into account), acceptability and coproduction constraints, revealed that it is possible to reach up to 95% of energy intake from plant-based foods.

The %PE was then constrained to gradually increased by 5% increments from the basal scenario (keeping the observed value of 65% of energy from plant-based foods but meeting nutritional and other constraints) to the final scenario (reaching the maximal value of 95% of energy from plant-based foods always allowing the satisfaction of constraints).

Modeled diet compositions across these scenarios are presented in Fig. 1. Progressive increase by increments of 5% in %PE was associated with a progressive decrease or a total removal of meat (ruminant, pork and poultry), dairy products, eggs, fat and dressing, fruit juices, prepared dishes/fast food, sweet and fat foods (SFF). On contrary, across scenarios, there was a progressive increase in dried fruit, legumes, soy-based products, vegetables, vegetable oil and whole-grain products. We observed a bell-shaped relationship for cereal, fruit, and beverages (fruit nectar, syrup, soda (with or without sugar, plant-based beverages (except soy-based), milk consumed with tea/coffee). Potatoes showed a bell- shaped distribution but a drastic increase in the 95%PE scenario. Of note, some food groups were increased as early as the basal scenario (65%PE) so as to correct the nutritional inadequacies of the observed diets (that did not comply with some nutritional constraints): beef, poultry, eggs, cereals, fast-food, fruit, legumes, whole grain products, oil, prepared dishes/fast food and SFF. Fish was stable across all scenario.

Nutrient contents of the diets and dietary indexes are shown in Table 2. The basal scenario (65%PE) under nutritional constraints led to an increase in energy intake (both from animal and plant-based foods and similar results for proteins). As a result of nutritional constraints, DHA (docosahexaenoic acid) + EPA (eicosapentaenoic acid), bioavailable iron, fibre and all micronutrient content of the diet were improved (from plus 1% for vitamin B9 to 21% for bioavailable iron). Then, the gradual increase in %PE (from 65 up to 95%) was associated with decreases in total and animal protein (− 28% and − 80% respectively) and an increase in plant protein (+ 72%). DHA and EPA intakes were stable across scenarios as well as bioavailable zinc and sodium.

**Table 2:** Nutritional and health indicators across scenarios of increase in % of energy from plant-based foods^1^.

	Obs	65% basal	*Δ65*_*%*_ _*vs. obs*_	70%	75%	80%	85%	90%	95%	*Δ*_*95%*_ _*vs. obs*_	*Δ*_*95%*_ _*vs. 65%*_
Nutrients											
EI (Kcal/d)	2001	2370	*18%*	2372	2461	2380	2370	2370	2375	*19%*	*0%*
EI from plant-based food (Kcal/d)	1415	1658	*17%*	1661	1846	1904	2016	2134	2256	*59%*	*36%*
EI from animal-based food (Kcal/d)	586	713	*22%*	711	615	475	355	236	119	*− 80%*	*− 83%*
EI from plant food (%)	71	70	*− 2%*	70	75	80	85	90	95	*34%*	*36%*
Protein intake (g/d)	91	107	*18%*	107	103	95	87	81	77	*− 15%*	*− 28%*
% EI from protein	18	18	*0%*	18	17	16	15	14	13	*− 28%*	*− 28%*
Plant protein (g/d)	29	37	*27%*	37	41	44	48	53	63	*118%*	*72%*
Animal protein (g/d)	62	70	*13%*	70	62	51	39	28	14	*− 78%*	*− 80%*
% Protein from plant-based food	31	34	*11%*	35	40	47	55	65	82	*164%*	*138%*
Vitamin B12 (µg/d)	6.5	7.09	*9%*	7.08	6.7	6.5	6.36	6.37	4	*− 38%*	*− 44%*
DHA + EPA (g/d)	0.44	0.50	*14%*	0.50	0.50	0.50	0.50	0.50	0.50	*14%*	*0%*
Selenium	81.34	87.53	*8%*	87.52	85.7	81.15	73.1	70.43	74.18	*− 9%*	*− 15%*
Potassium	3808	3560	*− 7%*	3561	3616	3652	3660	3838	4769	*25%*	*34%*
Vitamin B9	419.42	424.37	*1%*	424.54	438.64	467.6	500.5	574.62	719.88	*72%*	*70%*
Bioavailable zinc (mg/d)	3.3	3.41	*3%*	3.4	3.3	3.3	3.3	3.3	3.3	*0%*	*− 3%*
Bioavailable iron (mg/d)	1.7	2.06	*21%*	2.06	2.02	2.06	2.09	2.2	2.29	*35%*	*11%*
Calcium (mg/d)	1115	948	*− 15%*	948	948	947	947	947	950	*− 15%*	*0%*
Fibers (g/d)	23.35	30	*28%*	30	32.32	34.87	36.68	40.15	47.84	*105%*	*59%*
Sodium (mg/d)	2502	2294	*− 8%*	2294	2294	2294	2294	2294	2300	*− 8%*	*0%*
Indexes											
PANDiet	64.98	70.28	*8%*	70.32	72.11	77.92	81.24	81.12	81.97	*26%*	*17%*
PANDiet adequation subscore	78.86	93.51	*19%*	93.51	93.45	93.54	93.53	93.69	93.85	*19%*	*0%*
PANDiet moderation subscore	51.1	47.06	*− 8%*	47.12	50.77	62.3	68.95	68.56	70.08	*37%*	*9%*
cDQI	48.43	63.88	*32%*	63.89	64.56	65.83	56.55	55.75	58.7	*21%*	*− 8%*
pDQI	32.86	42.6	*30%*	42.59	42.45	43.75	43.33	42.4	44.58	*36%*	*5%*
aDQI	15.57	21.28	*37%*	21.29	22.11	22.07	13.22	13.35	14.12	*− 9%*	*− 34%*
PNNS-GS2	2.73	6.25	*129%*	6.25	6.73	7.25	6.75	6.75	6.75	*147%*	*8%*

Abbreviations: aDQI, animal diet quality index; cDQI, diet quality index; DHA, docosahexaenoic acid; EPA, eicosapentaenoic acid; PANDiet, Diet Quality Index Based on the Probability of Adequate Nutrient Intake; sPNNS-GS2: simplified Programme National Nutrition Santé guidelines score; Obs, observed diet; pDQI, plant diet quality index; PUFA, polyunsaturated fatty acids.

^1^Values are estimates for incremental 5% increases in the % of energy intake from plant-based foods. The basal scenario (65%) correspond to the modelled diet when the proportion of energy intake from plant-based foods is set at the observed value of proportion of energy intake from plant-based foods under nutritional, fish consumption limitation and coproducts constraints. Next scenarios increase plant-based foods energy from 65 up to 95%.

As expected, the basal scenario (65%PE) that corrected the nutritional inadequacies of the observed diet led to a healthier diet as reflected by an overall increase in PANDiet (+ 8%) and specifically of its adequation subscore. Similarly, the cDQI was improved (+ 32%) as well as each of its plant and animal subscores. Through scenarios of gradual %PE, the PANDiet gradually increased, until it reaches a plateau. Specifically, its adequation subscore was stable while its moderation subscore improved (+ 49%). As regards the cDQI, a small decrease was observed due to a decrease in aDQI. As regards the sPNNS-GS2, the basal scenario led to a strong increase in sPNNS-GS2 (+ 129%). Across scenario, gradual increase %PE led to increase in these both scores (+ 8 and + 22% respectively) with maximal values attained at around 80–85%.

The active constraints (i.e. limiting the model) in the basal scenario were, in descending order, EPA + DHA, energy intake, alpha-linolenic acid, saturated fatty acids, fiber, sodium, alpha-linoleic acid, and vitamin C. The active constraints in the 95% scenario were, in descending order, energy intake, bioavailable zinc, EPA + DHA, calcium, sodium, iodine, sugar without lactose, vitamin C and vitamin B12. Of note, vitamin B12 was limiting only in the last scenario (data not tabulated).

Environmental indicators for observed and optimized diets and each modelled scenario are showed in Table 3. Due to an increase in energy intake in the optimized diets, imposed by the energy requirements constraint (Table 1), the basal model scenario was associated with higher values compared to observed ones for GHGe, energy demand and land occupation, whatever the farming method. In the following scenarios, the gradual increase in energy from plant-based foods led to marked gradual decreases in all indicators, comparable whatever the farming method, around − 70% for GHGe, − 50% for energy demand and − 60% for land occupation between the final and initial.

**Table 3:** Environmental indicators for observed diet and trajectories of increase in proportion of energy intake from plant-based foods^1^.

	Obs	65%	*Δ*_*65% vs. obs*_	70%	75%	80%	85%	90%	95%	*Δ*_*95% vs. obs*_	*Δ*_*95% vs. 65%*_
100% conventional production											
GHGe (kgCO2eq/d)	4.06	4.57	*13%*	4.56	4.08	3.58	3.03	2.17	1.46	*− 64%*	*− 68%*
Energy demand (MJ/d)	18.14	19.43	*7%*	19.41	18.06	15.97	13.69	11.92	9.37	*− 48%*	*− 52%*
Land occupation (m^2^/d)	9.79	11.56	*18%*	11.55	10.57	9.54	8.33	6.11	4.48	*− 54%*	*− 61%*
100% organic production											
GHGe (kgCO2eq/d)	4.09	4.68	*14%*	4.67	4.13	3.61	3.03	2.14	1.44	*− 65%*	*− 69%*
Energy demand (MJ/d)	16.63	18.74	*13%*	18.71	17.4	15.52	13.51	11.58	9.54	*− 43%*	*− 49%*
Land occupation (m^2^/d)	13.35	15.74	*18%*	15.72	14.29	12.67	10.88	7.98	5.81	*− 56%*	*− 63%*

GHGe, greenhouse gas emissions; Obs, observed diet.

^1^Values are estimates for incremental 5% increases in the % of energy intake from plant-based foods. The basal scenario (65%) correspond to the modelled diet when the proportion of energy intake from plant-based foods is set at the observed value of proportion of energy intake from plant-based foods under nutritional, fish consumption limitation and coproducts constraints. Next scenarios increase plant-based foods energy from 65 up to 95%

**Figure 2:**
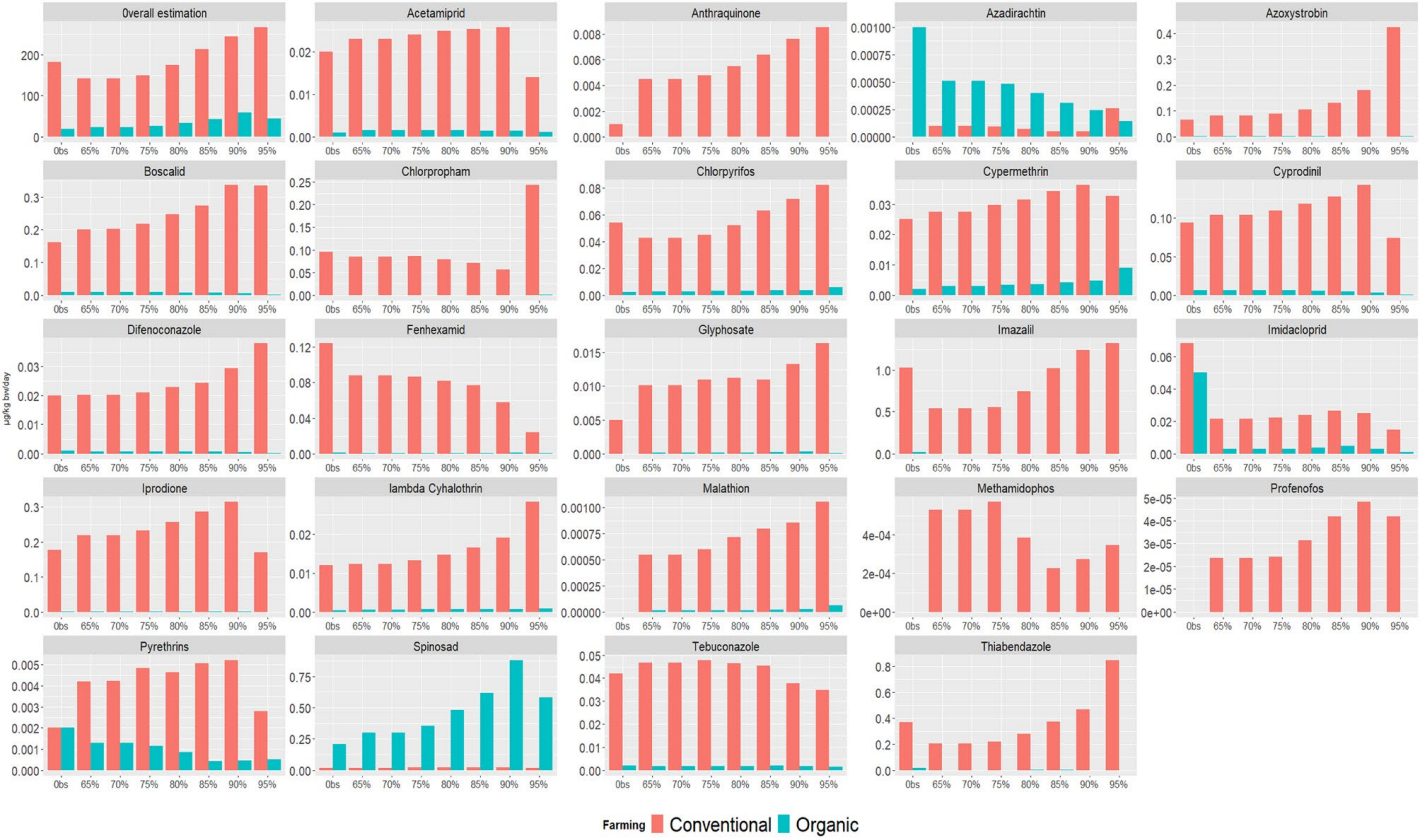
Estimated daily exposure to pesticide residues (μg/kg bw/day), in observed and modelled diets with gradual increase in proportion of energy intake from plant-based foods, according to 100%-conventional and 100%-organic modelling^1,2,3^. Abbreviations: ADI: acceptable daily intake; Obs, observed diet. ^1^ The basal scenario (65%) correspond to the modelled diet when the proportion of energy intake from plant-based foods is set at the observed value of proportion of energy intake from plant-based foods under nutritional, fish consumption limitation and coproducts constraints. Next scenarios increase plant-based foods energy from 65% up to 95%. ^2^ The overall estimation is calculated as the sum of individual exposure weighted by 1/DJA (without anthraquinone which has no ADI). ^3^ Natural pyrethrins and Spinosad are authorized in certified organic production.

Exposure to pesticide residues from plant-based foods are presented as 100% organic or 100% conventional for each scenario in Fig. 2. When modelling pesticide residues exposures, the increase in plant-based foods led to higher exposures to most of pesticides in the 100% conventional scenario, with some fluctuations depending on the structure of the modelled diet, conversely, 100% organic allowed to markedly limit exposure to synthetic pesticides. However, spinosad, which is approved in organic farming, increased. The exposure across scenarios are tabulated in Supplemental Table 2 and Supplemental Table 3 as % of the ADI. In relative value, compared to the observed situation, the synthetic indicator of exposure to pesticides increased in both farming systems (+ 46% in conventional and + 124% in organic), but values in organic were dramatically lower than in conventional (− 84% between the organic and conventional scenarios at 95%PE).

**Figure 3:**
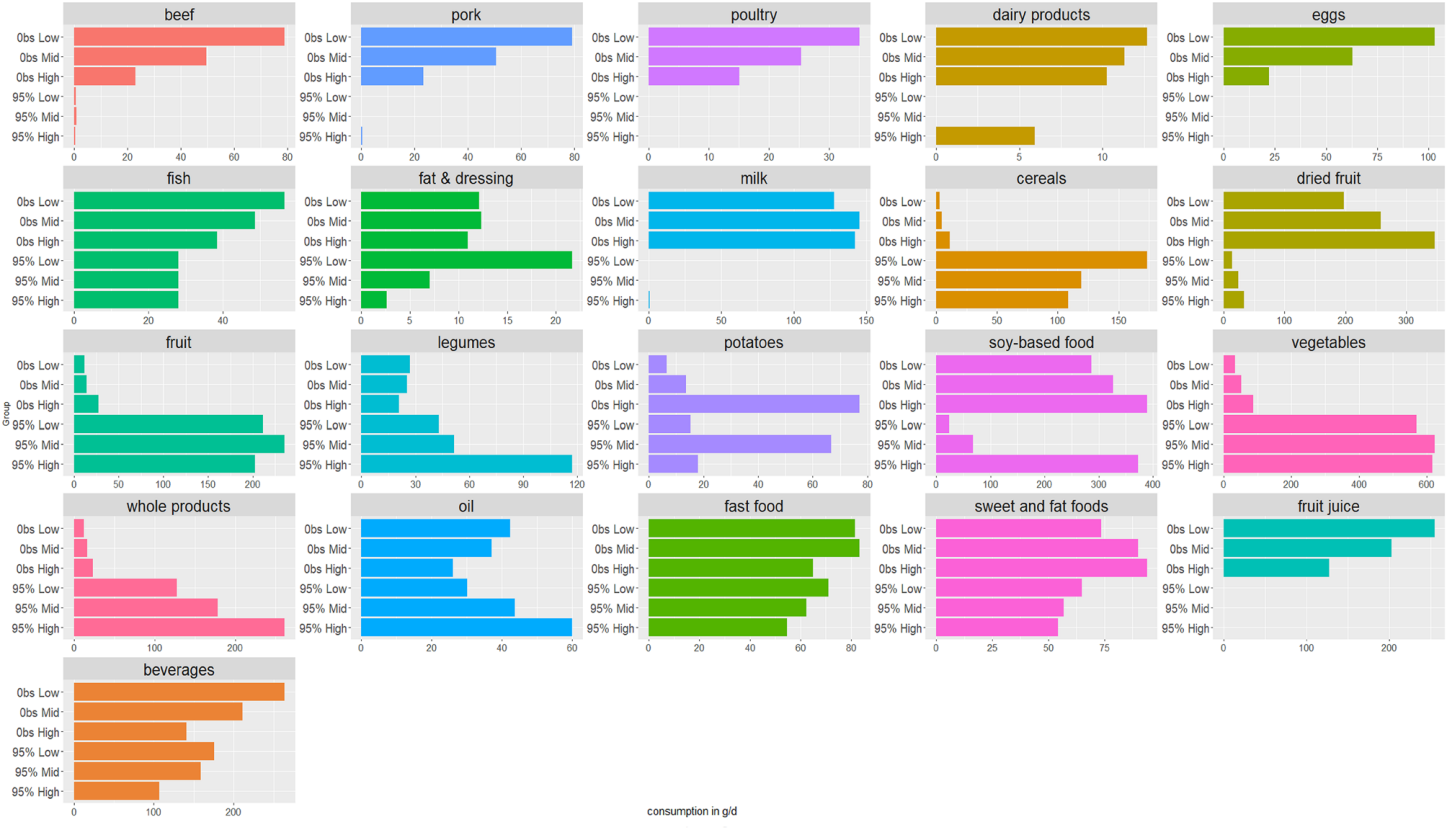
Variations in the composition (g/d) of the observed diet and 95% energy from plant food modelled diets according to observed level of plant food consumption^1,2^. Abbreviations: Obs, observed diet. SFF, sweet and fat foods. “Obs Low” corresponds to observed consumption in the group with at least 50% of energy from plant food at baseline. “Obs Mid” corresponds to observed consumption in the group with at least 65% of energy from plant food at baseline. “Obs High” corresponds to observed consumption in the group with at least 80% of energy from plant food at baseline. “95% Low” corresponds to the final scenario in the group with at least 50% of energy from plant food at baseline. “95% Mid” corresponds to the final scenario in the group with at least 65% of energy from plant food at baseline. “95% High” corresponds to the final scenario in the group with at least 80% of energy from plant food at baseline. ^1^Food group consumption (g/d) in the observed diets and in the 95%PE model according initial %PE. ^2^Vegetables include all vegetables and soups, fruit include fresh fruit, fruit in syrup and compote, dried fruit and seeds, fish include seafood, dairy product include yogurts, fresh cheese and cheese, potatoes include other tubers, cereals include breakfast cereal low in sugar, bread semolina, rice and pasta, sweet and fat foods include croissants, pastries, chocolate, biscuits, milky dessert, ice cream, honey and marmalade, cakes, chips, salted oilseeds, salted biscuits, beverages include fruit nectar, syrup, soda (with or without sugar), plant-based beverages (except soy-based), milk consumed with tea/coffee, fast-food include sandwich, prepared foods such as pizza, hamburger, ravioli, panini, salted pancake, etc., soy-based foods include tofu, soy meat substitute and soy yogurt, soy milk, and fats include fresh cream and butter.

A number of sensitivity analyses were conducted. The first method explore the influence of the observed level of energy intake from plant-based foods on the scenarios. Gradual optimized diets derived in subsamples with 50%PE, 65%PE, and 80%PE led to similar shapes of dietary trajectories. There were however some differences since the optimized consumptions of dried fruits and nuts, legumes, soy-based products, vegetables, and whole grain products increased in line with the baseline values of the %PE. Food group consumptions in the observed and optimized diets of the final scenario (95%PE) are presented in Fig. 3. The higher the %PE in the observed situation, the higher the optimized consumption of dried fruits and nuts, legumes, soy-based products, vegetables, and whole grain products.

The second sensitivity analyses modelled a gradual increase in plant proteins rather than in plant energy. The maximum contribution of plant proteins achievable for complying with the set of constraints was 80%. Consumptions in scenarios of gradual increase in plant proteins are shown in Supplemental Fig. 4. Findings were similar to those of the increase in %PE models but beef and milk decreased more rapidly while legumes increased more rapidly. In addition, sweet and fat product were higher in optimized diets. Compared to those of the increase in %PE models, findings were similar in terms of trends but maximum PANDiet was lower (76.47 vs. 81.97) (Supplemental Tables 4). Also, decrease in GHGe was stronger in conventional (1.01 vs. 1.46 kgCO2eq/d) and in organic 0.93 vs. 1.44 kgCO2eq/d) (Supplemental Table 5).

## Discussion

In this study evaluating a gradually increase in proportion of plant-based foods in the diet, we showed that it is possible to increase the caloric proportion of plant foods up to 95% (corresponding to 82% of protein from plant foods), without jeopardizing nutritional requirements in the French context of non-fortified foods. This increase in the proportion of plant-based foods is associated with a significant reduction in environmental pressures and, in particular of GHGe (about − 65%, in conventional and in organic scenario) as well as land occupation (-about − 55%, in conventional and in organic scenario). Although it has been shown in previous studies that a higher consumption of plant food is related to a higher exposure to pesticides, this is the first study to put it in the context of dietary changes for environmental sustainability. Nonetheless, compared to a 100% conventional diet, a 100% organic diet resulted in significantly lower exposure to pesticides residues (on average − 85%).

The most limiting nutrients that were stuck at their bounds (requirements or upper limits) in nearly all the optimized diets across scenarios were DHA + EPA, calcium, sodium, and bioavailable zinc.

Following previous work documenting a likely overestimated nutritional reference for zinc^25^, we selected a compromise between nutritional reference and deficiency threshold to set the constraint at the observed value to not over-shape the model. In spite of this release, the zinc constraint remained the most limiting in the basal scenario (65%PE). Sugars except lactose and sodium were also active constraints at the upper bound. As previously documented^56,57^, accounting for the bioavailability of iron and zinc using validated equations showed that such nutrients are key elements to consider in plant-based diets.

It should be noted that adequate nutrient intake can be achieved up to a scenario with 95% PE (or ≈ 80% protein from plant foods). This shows that a predominantly plant-based diet can provide adequate nutrient intake.

In that scenario, some nutrients from animal-based foods were critical, particularly zinc, EPA and DHA, calcium, iodine, and vitamin B12 and nutritional constraints were no longer achievable above the 95%PE scenario (mostly vitamin B12 and EPA + DHA constraints). Thus, our findings suggest the existence of levers for increase plant-foods in the diet without compromising nutritional quality.

Constraints to ensure nutrient requirements in the modelled diets resulted in an increase in the adequacy subscore (+ 19%) of PANDiet, from the first scenario, but this subscore then remained stable in the scenarios of gradual increases in plant foods. In contrast, the moderation subscore of PANDiet gradually improved.

Similarly, the cDQI improved significantly in the first scenario and then increased very slightly, and finally decreased. The plant component (pDQI) reached a plateau, while the animal component (aDQI) decreases with the gradual removal of animal-based foods. Overall, the quality of the diet is significantly improved with increasing plant foods in the diet and appears to peak around 80–85% of energy from plant foods. The association between the diet contribution of plant-based foods and diet quality^58^, estimated through holistic approaches such as dietary indexes, has been documented in the scientific literature^58^. However, data are relatively scarce, mostly focused on vegetarians and vegans diets in comparisons with meat-eaters through dietary indexes based on food group intakes rather than on nutrients intakes and requirements^59^.

Two recent studies have focused on the identification of the healthier plant to total protein ratio to be achieved while meeting nutritional references^60,61^. One of these studies focused only on nutritional aspects without reporting environmental pressures and reported an optimal ratio between 45 and 60%^61^. The second study documented that plant-based protein ratios could range from 15 to 80% without undermining the quality of diet^60^. However, as in our study, the optimized diets were different from the observed diets, and environmental pressures were diminished as the proportion of plant proteins increased. It is also worth noting that even though the modeling and population were different, the 80% plant protein ratio identified in the second study was very close to the value found (the model with 95% energy intake led to a 80% plant-based protein ratio) in our study. Based on observed data, we previously showed that a provegetarian score is positively associated with the PANDiet score reflecting the probability of adequacy to nutritional references^62^. Of note, we used the cDQI distinguishing the quality of foods from animal and plant origin^37^, which allows a better understanding of the combination of plant and animal foods that provide nutrients.

In terms of food consumption, the gradual increase in protein and energy from plant foods resulted to quite similar diets for both models. However, the models, as combinatory processes based on different objectives, led to some disparities, especially for foods with different protein contents. For example, for dairy products, the model aiming to reduce animal protein will favour milk that is less rich in protein than fresh dairy products. Besides, a salient point concerns the increase in exposure to pesticide residues associated with a diet rich in plant-based food. Indeed, fruit and vegetables are the food groups exhibiting the highest levels of pesticide residues, along with legumes and whole-grain cereals^27^ while animal foods are generally much less contaminated. Organic farming prohibits the use of synthetic pesticides and thus organically grown plant-based foods contain fewer and less often pesticide residues than their conventional counterparts thus allowing to reduce exposure to pesticides residues^63,64^. However, contaminations by remnant molecules are possible as the conversion towards organic farming is recent and some molecules are persistent^65^.

Of note exposure to individual compounds were mostly under ADI but it is now stated that exposure to low doses of mixture of pesticides residues may be harmful^66^.

As pesticide use also depends on crop types, the scenarios of gradual increase in plant-based foods led to increases or decreases in the total exposure. However, the overall food exposure indicator increased in both farming practices, but was six times more in conventional than in organic farming. All specific exposures were lower in organic than in conventional farming, except for the molecules which are authorized in organic farming, namely spinosad and pyrethrins. These findings are in line with those documented recently as regards the level of diet-related pesticides exposure according to different diets^28^.

Knowledge of the increased risk of disease associated with chronic exposure to pesticides, particularly in the occupational population, is growing^67–69^, but ad hoc studies should be conducted in the general population to better assess the potential risks associated with pesticide mixtures.

Consistent with the literature on observational data^13,14,70^ or modelled data using optimization algorithms^11–13^, the increase in the contribution of plant-based foods to diet was associated herein with lower environmental pressures. We hence obtained a 65% GHGe reduction for the final scenario with 95% of energy from plant-based foods compared to the observed situation, which also corresponds to the difference observed between omnivores and vegetarians^71^. This quantified reduction corresponds to the lower value of a vegetarian diet reported in the review by Aleksandrowicz et al.^13^ although the LCA were estimated at the farm level only in our study. We also obtained land use and energy demand decreases, which were of very similar extents whatever the mode of production. In the organic compared to the conventional production farming system, land occupation was higher and energy demand was lower, but the differences according farming practices were attenuated across scenario.

However, diets that are much higher in plant-based foods than in animal foods can raise agronomic issues such as the alternative use of permanent grasslands in case of reduction in livestock farming. In particular, because some areas, especially mountainous ones, are ideal for livestock farming. It should also be noted that carbon sequestration is not sufficient to offset beef emissions, in particular because the carbon sinks are eventually saturated^72^*.* In that context, some strategies, although insufficient at present, have been proposed to mitigate gas emissions by ruminants including animal and feed management, diet formulation and rumen manipulation^73^. Most of the soybeans used for animal feed in France and Europe are imported from Latin America, which contributes significantly to deforestation in these countries^74^. Despite public policy efforts^75^, this type of soy production is unsustainable (because it is transported from a far distance) and cannot be part of a sustainable food system. The high consumption of soy products identified in the present study would therefore require a reallocation of soybean production locally and appropriate and sustainable management practices to allow for sustainable soy production for human consumption^76^.

Overall, our results are coherent with the literature comparing GHG emissions from observed diets more or less rich in animal products, with lower emissions for diets richer in plant-based foods^13,14^, although such observed diets do not necessarily meet the nutritional requirements.

The final scenario (95%PE) had similarities to the 2030 scenario modelled in the Netherlands, except that it included more fruits and vegetables, less dairy products, and significantly more soy-based products^77^. Similar to our findings, fish was still needed to ensure EPA + DHA intakes. While the LCAs used herein are based on the farm perimeter, GHGe were comparable in this study and ours. We recently conducted a diet optimization model study showing that it is possible to reduce GHGs by 50% in the NutriNet-Santé population without eliminating all animal-based foods^17^. The present study demonstrated that, under nutritional constraints, it is possible to further reduce GHGs by up to − 65% by eliminating almost all animal products while meeting nutritional requirements.

The acceptability of these diets is questionable, especially since very high fiber intake may cause intestinal discomfort for certain populations^78^*.* However, the aim of this work is purely cognitive, that is, we study the consequences of the degree of vegetation without making recommendations on the degree to be achieved.

Our study has limitations which should be highlighted. First, composition data in terms of amino acids were not available to better characterize the adequacy of indispensable amino acid beyond that of protein (nitrogen). However, some literature argues that in countries without protein insufficiency, these could not be a limiting issue^79^. Second, life cycle assessments were restricted to the production stage because they were not available in the organic system for the entire system. Although the production stage is the main source of pressure, it would be interesting to be able to consider the pressures up to the plate especially for GHGe and energy demand. In addition, it is well documented that LCA misestimates some ecosystem services in particular for agroecological practices^80^. The environmental analysis encompassed three major indicators^81^ which, although important, are not sufficient to conduct a comprehensive analysis in particular as regards blue water and biodiversity loss. Consumption data were collected in 2014 and therefore do not accurately reflect current eating habits. The same applies to environmental and pesticide residue data. Data will need to be updated to allow to examine how models may evolve. Finally, participants were volunteers, and therefore probably more concerned by nutritional issues. Thus, the observed diet (starting point of the optimization) was already richer in plant-based foods than that of the general population but in the secondary analysis showed that similar findings were observed even in a group with low plant-based food in the observed situation.

Nonetheless, the strengths of our study are multiple. We used a multicriteria approach when modeling diets (by considering nutritional requirements, cultural acceptability and coproduction links) and when evaluating diet impacts (on both health, environment and safety indicators), by moreover distinguishing between the organic and standard/conventional farming systems. We have considered the coproduction links between beef and milk, but it would have been interesting to consider the link between oil and oilcake for rapeseed, for example, but data are lacking to estimate these factors. Finally, the list of foods was highly detailed, allowing to select those with the most nutritional interest, and a wide set of nutritional reference values were used, including bioavailability for zinc and iron, which may be an issue in plant-based diets.

## Conclusion

This study documented in an original way the possibility to increase the plant part of the diet up to an extreme level while providing nutritionally adequate diets. This leads to a drastic reduction of some environmental indicators, in particular land occupation and GHGe, and is therefore an important lever in the framework of the climate strategy. However, the increase in plant-based foods consumptions leads to a substantial increase in exposure to pesticide residues, in particular for farming practice using synthetic pesticides, which should be thoroughly characterized in terms of risk. The increase in the proportion of plant-based foods in the diet, which is beneficial for both human health and the planet, must therefore be accompanied by appropriate policies allowing a wide access to plant products with a low content of pesticide residues (e.g. organic products).

### Supplementary Information

Supplementary Information.

## Data Availability

Script and data would be available upon reasonable request to the corresponding author emmanuelle.kesse-guyot@inrae.fr. Researchers from public institutions can submit a collaboration request including information on the institution and a brief description of the project to collaboration@etude-nutrinet-sante.fr. All requests will be reviewed by the steering committee of the NutriNet-Santé study. A financial contribution may be requested. If the collaboration is accepted, a data access agreement will be necessary and appropriate authorizations from the competent administrative authorities may be needed. In accordance with existing regulations, no personal data will be accessible.

## Abbreviations

DHA: Docosahexaenoic acid
DQI: Diet quality index
EPA: Eicosapentaenoic acid
FFQ: Food frequency questionnaire
GHGe: Greenhouse gas emissions
PE: Energy from plant food
PNNS-GS: Programme National Nutrition Santé-guidelines score
SFF: Sweet and fat foods
