# Supplementary material for: Environmental pressures and pesticide exposure associated with an increase in the share of plant-based foods in the diet
Source: Sci Rep. 2023 Nov 7;13:19317. doi: 10.1038/s41598-023-46032-z (PMC10630347; doi:10.1038/s41598-023-46032-z)
Supplement: Supplementary file 1 — Supplementary Information. [file 41598_2023_46032_MOESM1_ESM.docx]

**Supplemental Material**

***Iron bioavailability***

For iron bioavailability, we considered heme and non-heme iron.

The rate of absorption for heme iron was calculated as (1):

$$\text{Log Absorption }\left( \text{\%} \right)\text{= 1.9897 – 0.3092 × log (SF) }$$

where SF is serum ferritin (μg/L). We considered a stringent situation by setting serum ferritin at 15 mg/L (2).

The rate of absorption for non-heme iron was calculated as (3):

$$\text{Ln }\text{ }\left( \text{\%} \right)\text{= }\text{6.294 – 0.709 ln }\left( \text{SF} \right)\text{+ 0.119 ln }\left( \text{Vit}\text{C} \right)\text{+ 0.006 ln }\left( \text{MFP + 0.1} \right)\text{ } \text{-0.055}\ln\left( \text{T+0.1} \right)\text{-0.247}\ln\left( \text{P}\text{hy} \right)\text{-0.137}\ln\left( \text{Ca} \right)\text{-0.083 ln}\text{ }\text{(NH}\text{I}\text{)}$$

$\text{Ln Absorption }\left( \text{\%} \right)\text{= }\text{6.294 – 0.709 }\text{ln}\text{ }\left( \text{SF} \right)\text{+ 0.119 ln }\left( \text{Vit}\text{C} \right)\text{+ 0.006 ln}\text{ }\left( \text{MFP + 0.1} \right)\text{ } \text{-0.055}\ln\left( \text{T+0.1} \right) \text{-0.247}\ln\left( \text{P}\text{hy} \right)\text{-0.137}\ln\left( \text{Ca} \right)\text{-0.083 ln}\text{ }\text{(NH}\text{I}\text{)}$

where SF is serum ferritin (μg/L) which was also set at 15 mg/L, VitC is vitamin C intake (mg), MFP corresponds to consumption of meat, fish, and poultry (g), T is tea intake (as number of cups), Phy is phytate intake (mg), Ca is calcium intake (mg), and NHI is non-heme iron intake (mg).

***Zinc*** ***bioavailability***

For zinc bioavailability, we used the equation developed and updated by Miller and al. (4):

$$\text{BZ= 0.5 × }\left[ \text{0.033 ×}\left( \text{1 +}\frac{\mathrm{DPhy}}{\text{0.68}} \right)\text{+ 0.091 + }\text{DZ}\text{ -} \sqrt{\left( \text{0.033 ×}\left( \text{1 +}\frac{\mathrm{DPhy}}{\text{0.68}} \right)\text{+ 0.091 + }\text{DZ } \right)^{\text{2}}\text{- 4 × 0.091 × }\text{DZ }} \right]$$

where BZ is bioavailable zinc (mmol), DZ is dietary zinc (mmol) and DPhy corresponds to dietary (mmol).

1. Hallberg L, Hulthén L. Prediction of dietary iron absorption: an algorithm for calculating absorption and bioavailability of dietary iron. Am J Clin Nutr. 2000 May;71(5):1147–60.

2. Hallberg L, Brune M, Rossander L. Iron absorption in man: ascorbic acid and dose-dependent inhibition by phytate. Am J Clin Nutr. 1989 Jan;49(1):140–4.

3. Armah SM, Carriquiry A, Sullivan D, Cook JD, Reddy MB. A complete diet-based algorithm for predicting nonheme iron absorption in adults. J Nutr. 2013 Jul;143(7):1136–40.

4. Miller LV, Krebs NF, Hambidge KM. A mathematical model of zinc absorption in humans as a function of dietary zinc and phytate. J Nutr. 2007 Jan;137(1):135–41.

**Supplemental Table 1: Baseline characteristics by subgroup, n=29,413, NutriNet-Santé, 2014**

|  | **Men** | **Premenopausal women** | **Menopausal women** | **Average individual** |
| --- | --- | --- | --- | --- |
| **N** | 7,416 | 9,710 | 12,287 | 29,413 |
| **Age (y)** | 58.47 (13.03) | 39.27 (9.59) | 61.80 (6.99) | 54.49 (14.12) |
| **Body mass index (kg/m²)** | 25.30 (4.00) | 23.13 (4.66) | 24.38 (4.77) | 24.52 (4.46) |
| **Education (%)** |  |  |  |  |
| < High-school diploma | 26.4 | 8.6 | 28.2 | 22.37 |
| High school diploma | 12.4 | 12.6 | 17.6 | 13.75 |
| Postgraduate | 61.2 | 78.9 | 54.2 | 63.87 |
| **Occupation (%)** |  |  |  |  |
| Unemployed | 2.9 | 6.4 | 3.0 | 3.79 |
| Retired | 53.1 | 2.0 | 55.3 | 40.84 |
| Employee, manual worker | 7.5 | 24.3 | 10.5 | 12.46 |
| Intermediate professions | 9.8 | 23.4 | 10.9 | 13.49 |
| Managerial staff, intellectual profession | 22.6 | 31.6 | 11.8 | 22.17 |
| Never employed | 2.0 | 10.3 | 7.0 | 5.36 |
| Self-employed, farmer | 2.1 | 2.0 | 1.4 | 1.9 |
| **Physical activity level (%)** |  |  |  |  |
| Missing data | 9.1 | 11.3 | 11.4 | 18.69 |
| Low | 16.8 | 24.4 | 16.6 | 10.23 |
| Moderate | 32.5 | 41.0 | 35.1 | 35.84 |
| High | 41.6 | 23.3 | 36.9 | 35.25 |
| **Tobacco status (%)** |  |  |  |  |
| Never smoker | 38.8 | 56.3 | 48.8 | 45.68 |
| Former smoker | 51.9 | 28.6 | 43.0 | 43.84 |
| Current smoker | 9.3 | 15.1 | 8.3 | 10.49 |
| Cost of the diet (€/d) | 8.59 (3.15) | 7.89 (2.91) | 6.79 (2.71) | 7.7 (2.99) |

^1^ Values are means ± SD or percent, as appropriate

**Supplemental Table 2: Exposure to pesticide residues in organic and conventional farming according to the scenarios ^1^**

| **Conventional** **system** | **Obs** | **65%**  **basal** | **70%** | **75%** | **80%** | **85%** | **90%** | **95%** | **ADI**  **(mg/kg bw/d)** | **Indivdiual ADI (mg/d) ^2^** |
| --- | --- | --- | --- | --- | --- | --- | --- | --- | --- | --- |
| Synthetic indicator ^3^ | 182.76 | 141.55 | 141.61 | 149.15 | 175.08 | 212.87 | 245.07 | 267.35 |  |  |
| Acetamiprid | 0.020 | 0.023 | 0.023 | 0.024 | 0.025 | 0.025 | 0.026 | 0.014 | 0.025 | 1.765 |
| Anthraquinone | 0.001 | 0.004 | 0.004 | 0.005 | 0.005 | 0.006 | 0.008 | 0.009 |  |  |
| Azadirachtin | 0.000 | 0.000 | 0.000 | 0.000 | 0.000 | 0.000 | 0.000 | 0.000 | 0.1 | 7.06 |
| Azoxystrobin | 0.066 | 0.082 | 0.082 | 0.088 | 0.106 | 0.132 | 0.182 | 0.424 | 0.2 | 14.12 |
| Boscalid | 0.162 | 0.202 | 0.202 | 0.217 | 0.247 | 0.274 | 0.336 | 0.336 | 0.04 | 2.824 |
| Chlorpropham | 0.095 | 0.084 | 0.084 | 0.086 | 0.079 | 0.070 | 0.057 | 0.243 | 0.05 | 3.53 |
| Chlorpyrifos | 0.054 | 0.043 | 0.043 | 0.045 | 0.052 | 0.064 | 0.072 | 0.082 | 0.001 | 0.0706 |
| Cypermethrin | 0.025 | 0.028 | 0.028 | 0.030 | 0.032 | 0.034 | 0.036 | 0.033 | 0.05 | 3.53 |
| Cyprodinil | 0.094 | 0.104 | 0.104 | 0.110 | 0.119 | 0.128 | 0.142 | 0.074 | 0.03 | 2.118 |
| Difenoconazole | 0.020 | 0.020 | 0.020 | 0.021 | 0.023 | 0.024 | 0.029 | 0.038 | 0.01 | 0.706 |
| Fenhexamid | 0.124 | 0.088 | 0.088 | 0.086 | 0.081 | 0.077 | 0.057 | 0.024 | 0.2 | 14.12 |
| Glyphosate | 0.005 | 0.010 | 0.010 | 0.011 | 0.011 | 0.011 | 0.013 | 0.016 | 0.5 | 35.3 |
| Imazalil | 1.026 | 0.536 | 0.536 | 0.556 | 0.744 | 1.023 | 1.239 | 1.317 | 0.025 | 1.765 |
| Imidacloprid | 0.068 | 0.021 | 0.021 | 0.022 | 0.024 | 0.026 | 0.025 | 0.015 | 0.06 | 4.236 |
| Iprodione | 0.177 | 0.218 | 0.218 | 0.233 | 0.257 | 0.287 | 0.314 | 0.170 | 0.02 | 1.412 |
| Malathion | 0.000 | 0.001 | 0.001 | 0.001 | 0.001 | 0.001 | 0.001 | 0.001 | 0.03 | 2.118 |
| Methamidophos | 0.000 | 0.001 | 0.001 | 0.001 | 0.000 | 0.000 | 0.000 | 0.000 | 0.001 | 0.0706 |
| Profenofos | 0.000 | 0.000 | 0.000 | 0.000 | 0.000 | 0.000 | 0.000 | 0.000 | 0.03 | 2.118 |
| Pyrethrins | 0.002 | 0.004 | 0.004 | 0.005 | 0.005 | 0.005 | 0.005 | 0.003 | 0.04 | 2.824 |
| Spinosad | 0.016 | 0.020 | 0.020 | 0.021 | 0.022 | 0.024 | 0.025 | 0.016 | 0.024 | 1.6944 |
| Tebuconazole | 0.042 | 0.047 | 0.047 | 0.048 | 0.046 | 0.045 | 0.038 | 0.035 | 0.03 | 2.118 |
| Thiabendazole | 0.367 | 0.207 | 0.207 | 0.217 | 0.278 | 0.373 | 0.467 | 0.844 | 0.1 | 7.06 |
| lambda_Cyhalothrin | 0.012 | 0.012 | 0.012 | 0.013 | 0.015 | 0.017 | 0.019 | 0.028 | 0.0025 | 0.1765 |
| **Organic system** | **Obs** | **65%**  **basal** | **70%** | **75%** | **80%** | **85%** | **90%** | **95%** | **ADI**  **(mg/kg bw/d)** | **Indivdiual ADI (mg/d)^1^** |
| Synthetic indicator | 19.53 | 23.32 | 23.35 | 26.93 | 34.48 | 42.81 | 58.40 | 43.78 | 25.42 |  |
| Acetamiprid | 0.001 | 0.002 | 0.002 | 0.002 | 0.001 | 0.001 | 0.001 | 0.001 | 0.025 | 1.765 |
| Anthraquinone | 0.000 | 0.000 | 0.000 | 0.000 | 0.000 | 0.000 | 0.000 | 0.000 |  |  |
| Azadirachtin | 0.001 | 0.001 | 0.001 | 0.000 | 0.000 | 0.000 | 0.000 | 0.000 | 0.1 | 7.06 |
| Azoxystrobin | 0.001 | 0.001 | 0.001 | 0.001 | 0.001 | 0.001 | 0.001 | 0.001 | 0.2 | 14.12 |
| Boscalid | 0.009 | 0.008 | 0.008 | 0.008 | 0.008 | 0.007 | 0.006 | 0.002 | 0.04 | 2.824 |
| Chlorpropham | 0.000 | 0.000 | 0.000 | 0.000 | 0.000 | 0.000 | 0.000 | 0.001 | 0.05 | 3.53 |
| Chlorpyrifos | 0.002 | 0.003 | 0.003 | 0.003 | 0.003 | 0.003 | 0.004 | 0.006 | 0.001 | 0.0706 |
| Cypermethrin | 0.002 | 0.003 | 0.003 | 0.003 | 0.004 | 0.004 | 0.005 | 0.009 | 0.05 | 3.53 |
| Cyprodinil | 0.006 | 0.006 | 0.006 | 0.006 | 0.005 | 0.004 | 0.003 | 0.000 | 0.03 | 2.118 |
| Difenoconazole | 0.001 | 0.001 | 0.001 | 0.001 | 0.001 | 0.001 | 0.001 | 0.000 | 0.01 | 0.706 |
| Fenhexamid | 0.001 | 0.001 | 0.001 | 0.001 | 0.001 | 0.001 | 0.001 | 0.000 | 0.2 | 14.12 |
| Glyphosate | 0.000 | 0.000 | 0.000 | 0.000 | 0.000 | 0.000 | 0.000 | 0.000 | 0.5 | 35.3 |
| Imazalil | 0.019 | 0.001 | 0.001 | 0.001 | 0.001 | 0.002 | 0.001 | 0.001 | 0.025 | 1.765 |
| Imidacloprid | 0.050 | 0.003 | 0.003 | 0.003 | 0.004 | 0.005 | 0.003 | 0.001 | 0.06 | 4.236 |
| Iprodione | 0.001 | 0.001 | 0.001 | 0.001 | 0.001 | 0.001 | 0.001 | 0.000 | 0.02 | 1.412 |
| Malathion | 0.000 | 0.000 | 0.000 | 0.000 | 0.000 | 0.000 | 0.000 | 0.000 | 0.03 | 2.118 |
| Methamidophos | 0.000 | 0.000 | 0.000 | 0.000 | 0.000 | 0.000 | 0.000 | 0.000 | 0.001 | 0.0706 |
| Profenofos | 0.000 | 0.000 | 0.000 | 0.000 | 0.000 | 0.000 | 0.000 | 0.000 | 0.03 | 2.118 |
| Pyrethrins | 0.002 | 0.001 | 0.001 | 0.001 | 0.001 | 0.000 | 0.000 | 0.001 | 0.04 | 2.824 |
| Spinosad | 0.210 | 0.301 | 0.301 | 0.353 | 0.479 | 0.615 | 0.877 | 0.581 | 0.024 | 1.6944 |
| Tebuconazole | 0.002 | 0.002 | 0.002 | 0.002 | 0.002 | 0.002 | 0.002 | 0.001 | 0.03 | 2.118 |
| Thiabendazole | 0.017 | 0.001 | 0.001 | 0.001 | 0.001 | 0.002 | 0.001 | 0.000 | 0.1 | 7.06 |
| lambda_Cyhalothrin | 0.000 | 0.001 | 0.001 | 0.001 | 0.001 | 0.001 | 0.001 | 0.001 | 0.0025 | 0.1765 |

Abbreviations: ADI, acceptable daily intake

^1^ Natural Pyrethrins and Spinosad are authorized in certified organic production.

^2^ ADI in mg per day for a human of 70.6kg

^3^ The overall estimation is calculated as the sum of individual exposure weighted by 1/DJA (without anthraquinone which has no ADI)

**Supplemental Table 3: Exposure to pesticide residues in organic and conventional farming as Percentage of the ADI according to the scenarios**

| **Conventional system** | **Obs** | **65%** | **70%** | **75%** | **80%** | **85%** | **90%** | **95%** |
| --- | --- | --- | --- | --- | --- | --- | --- | --- |
| Acetamiprid | 1 | 1 | 1 | 1 | 1 | 1 | 1 | 1 |
| Anthraquinone |  |  |  |  |  |  |  |  |
| Azadirachtin | 1 | 0 | 0 | 0 | 0 | 0 | 0 | 0 |
| Azoxystrobin |  | 1 | 1 | 1 | 1 | 1 | 1 | 3 |
| Boscalid | 0 | 7 | 7 | 8 | 9 | 10 | 12 | 12 |
| Chlorpropham | 0 | 2 | 2 | 2 | 2 | 2 | 2 | 7 |
| Chlorpyrifos | 6 | 60 | 60 | 64 | 74 | 90 | 102 | 117 |
| Cypermethrin | 3 | 1 | 1 | 1 | 1 | 1 | 1 | 1 |
| Cyprodinil | 76 | 5 | 5 | 5 | 6 | 6 | 7 | 3 |
| Difenoconazole | 1 | 3 | 3 | 3 | 3 | 3 | 4 | 5 |
| Fenhexamid | 4 | 1 | 1 | 1 | 1 | 1 | 0 | 0 |
| Glyphosate | 3 | 0 | 0 | 0 | 0 | 0 | 0 | 0 |
| Imazalil | 1 | 30 | 30 | 31 | 42 | 58 | 70 | 75 |
| Imidacloprid | 0 | 1 | 1 | 1 | 1 | 1 | 1 | 0 |
| Iprodione | 58 | 15 | 15 | 16 | 18 | 20 | 22 | 12 |
| Malathion | 2 | 0 | 0 | 0 | 0 | 0 | 0 | 0 |
| Methamidophos | 13 | 1 | 1 | 1 | 1 | 0 | 0 | 0 |
| Profenofos | 0 | 0 | 0 | 0 | 0 | 0 | 0 | 0 |
| Pyrethrins | 1 | 0 | 0 | 0 | 0 | 0 | 0 | 0 |
| Spinosad | 0 | 1 | 1 | 1 | 1 | 1 | 1 | 1 |
| Tebuconazole | 0 | 2 | 2 | 2 | 2 | 2 | 2 | 2 |
| Thiabendazole | 1 | 3 | 3 | 3 | 4 | 5 | 7 | 12 |
| lambda_Cyhalothrin | 2 | 7 | 7 | 8 | 8 | 9 | 11 | 16 |
| **Organic system** | **Obs** | **65%** | **70%** | **75%** | **80%** | **85%** | **90%** | **95%** |
| Acetamiprid | 0 | 0 | 0 | 0 | 0 | 0 | 0 | 0 |
| Anthraquinone |  |  |  |  |  |  |  |  |
| Azadirachtin | 0 | 0 | 0 | 0 | 0 | 0 | 0 | 0 |
| Azoxystrobin | 0 | 0 | 0 | 0 | 0 | 0 | 0 | 0 |
| Boscalid | 0 | 0 | 0 | 0 | 0 | 0 | 0 | 0 |
| Chlorpropham | 0 | 0 | 0 | 0 | 0 | 0 | 0 | 0 |
| Chlorpyrifos | 3 | 4 | 4 | 5 | 5 | 5 | 8 | 4 |
| Cypermethrin | 0 | 0 | 0 | 0 | 0 | 0 | 0 | 0 |
| Cyprodinil | 0 | 0 | 0 | 0 | 0 | 0 | 0 | 0 |
| Difenoconazole | 0 | 0 | 0 | 0 | 0 | 0 | 0 | 0 |
| Fenhexamid | 0 | 0 | 0 | 0 | 0 | 0 | 0 | 0 |
| Glyphosate | 0 | 0 | 0 | 0 | 0 | 0 | 0 | 0 |
| Imazalil | 1 | 0 | 0 | 0 | 0 | 0 | 0 | 0 |
| Imidacloprid | 1 | 0 | 0 | 0 | 0 | 0 | 0 | 0 |
| Iprodione | 0 | 0 | 0 | 0 | 0 | 0 | 0 | 0 |
| Malathion | 0 | 0 | 0 | 0 | 0 | 0 | 0 | 0 |
| Methamidophos | 0 | 0 | 0 | 0 | 0 | 0 | 0 | 0 |
| Profenofos | 0 | 0 | 0 | 0 | 0 | 0 | 0 | 0 |
| Pyrethrins | 0 | 0 | 0 | 0 | 0 | 0 | 0 | 0 |
| Spinosad | 12 | 18 | 21 | 28 | 36 | 52 | 34 | 18 |
| Tebuconazole | 0 | 0 | 0 | 0 | 0 | 0 | 0 | 0 |
| Thiabendazole | 0 | 0 | 0 | 0 | 0 | 0 | 0 | 0 |
| lambda_Cyhalothrin | 0 | 0 | 0 | 0 | 0 | 0 | 0 | 0 |

Abbreviations: ADI, acceptable daily intake; Obs, observed diet

^1^ Natural Pyrethrins and Spinosad are authorized in certified organic production.

**Supplemental Table 4: Nutritional and health indicators across scenario of increase in proportion of proteins from plant food**

|  | **Obs** | **30%**  **basal** | ***Δ_30% vs. obs_*** | **40%** | **50%** | **60%** | **70%** | **80%** | ***Δ_80% vs. obs_*** | ***Δ_80% vs. 30%_*** |
| --- | --- | --- | --- | --- | --- | --- | --- | --- | --- | --- |
| **Nutrients** |  |  |  |  |  |  |  |  |  |  |
| EI (Kcal/d) | 2001 | 2370 | *18%* | 2382 | 2370 | 2370 | 2370 | 2447 | *22%* | *3%* |
| EI from plant-based food (Kcal/d) | 1415 | 1658 | *17%* | 1749 | 1850 | 1957 | 2058 | 2254 | *59%* | *36%* |
| EI from animal-based food (Kcal/d) | 586 | 713 | *22%* | 633 | 520 | 414 | 312 | 193 | *-67%* | *-73%* |
| EI from plant food (%) | 71 | 70 | *-1%* | 73 | 78 | 83 | 87 | 92 | *30%* | *32%* |
| Protein intake (g/d) | 91 | 107 | *18%* | 103 | 92 | 84 | 78 | 78 | *-14%* | *-27%* |
| % EI from protein | 18 | 18 | *-1%* | 17 | 15 | 14 | 13 | 13 | *-30%* | *-29%* |
| Plant protein (g/d) | 29 | 37 | *29%* | 41 | 46 | 50 | 55 | 62 | *117%* | *69%* |
| Animal protein (g/d) | 62 | 70 | *13%* | 62 | 46 | 34 | 23 | 16 | *-75%* | *-78%* |
| % Protein from plant-based food | 31 | 34 | *10%* | 40 | 50 | 60 | 70 | 80 | *154%* | *132%* |
| Vitamin B12 (µg/d) | 6.50 | 7.10 | *9%* | 6.70 | 6.49 | 6.16 | 5.91 | 4.00 | *-38%* | *-44%* |
| DHA+EPA (g/d) | 0.44 | 0.5 | *14%* | 0.5 | 0.5 | 0.5 | 0.5 | 0.5 | *14%* | *0%* |
| Selenium | 81.34 | 87.71 | *8%* | 85.73 | 78.11 | 73.18 | 71.44 | 73.96 | *-9%* | *-16%* |
| Potassium | 3808 | 3567 | *-6%* | 3604 | 3634 | 3674 | 3734 | 4042 | *6%* | *13%* |
| Vitamin B9 | 419.42 | 425.30 | *1%* | 444.26 | 494.00 | 572.30 | 632.83 | 655.93 | *56%* | *54%* |
| Bioavailable zinc (mg/d) | 3.3 | 3.41 | *3%* | 3.30 | 3.30 | 3.30 | 3.30 | 3.30 | *0%* | *-3%* |
| Bioavailable iron (mg/d) | 1.7 | 2.06 | *21%* | 2.02 | 2.06 | 2.06 | 2.12 | 2.30 | *35%* | *11%* |
| Calcium (mg/d) | 1115 | 950 | *-15%* | 950 | 950 | 950 | 950 | 950 | *-15%* | *0%* |
| Fibers (g/d) | 23.35 | 30.00 | *28%* | 32.55 | 36.10 | 38.24 | 41.17 | 47.23 | *102%* | *57%* |
| Sodium (mg/d) | 2502 | 2300 | *-8%* | 2300 | 2300 | 2300 | 2300 | 2300 | *-8%* | *0%* |
| **Indexes** |  |  |  |  |  |  |  |  |  |  |
| PANDiet | 64.98 | 70.28 | *8%* | 72.45 | 73.59 | 76.96 | 78.58 | 76.47 | *18%* | *9%* |
| PANDiet adequation subscore | 78.86 | 93.51 | *19%* | 93.36 | 93.55 | 93.60 | 93.57 | 93.60 | *19%* | *0%* |
| PANDiet moderation subscore | 51.1 | 47.06 | *-8%* | 51.54 | 53.64 | 60.32 | 63.58 | 59.34 | *16%* | *26%* |
| pDQI | 32.86 | 42.60 | *30%* | 42.17 | 43.05 | 41.60 | 40.94 | 42.76 | *30%* | *0%* |
| aDQI | 15.57 | 21.28 | *37%* | 22.04 | 19.05 | 17.92 | 17.54 | 14.02 | *-10%* | *-34%* |
| cDQI | 48.43 | 63.88 | *32%* | 64.22 | 62.10 | 59.51 | 58.48 | 56.79 | *17%* | *-11%* |
| sPNNS-GS2 | 2.73 | 6.25 | *129%* | 7.25 | 7.25 | 7.25 | 7.25 | 6.30 | *131%* | *1%* |

Abbreviations: aDQI, animal diet quality index; cDQI, diet quality index; PANDiet, Diet Quality Index Based on the Probability of Adequate Nutrient Intake; sPNNS-GS2: simplified Programme National Nutrition Santé guidelines score; Obs, observed diet; pDQI, plant diet quality index; PUFA, polyunsaturated fatty acids;

^1 1^Values are estimates for incremental 10% increases in the % of protein intake from plant-based foods. The basal scenario (30%) corresponds to the modelled diet when the proportion of energy intake from plant-based foods is set at the observed value of proportion of protein intake from plant-based foods under nutritional, fish consumption limitation and coproducts constraints. Next scenarios increase plant-based foods protein from 30% up to 80%.

**Supplemental Table 5: Environmental indicators for observed diet and trajectories of increase in proportion of protein intake from plant food**

|  | **Obs** | **30%**  basal | *Δ_30% vs. obs_* | **40%** | **50%** | **60%** | **70%** | **80%** | ***Δ_80% vs. obs_*** | ***Δ_80% vs. 30%_*** |
| --- | --- | --- | --- | --- | --- | --- | --- | --- | --- | --- |
| 100% conventional production | |  |  |  |  |  |  |  |  |  |
| GHGe (kgCO2eq/d) | 4.06 | 4.57 | *13* | 4.03 | 3.47 | 2.65 | 1.65 | 1.01 | *-75* | *-78* |
| Energy demand (MJ/d) | 18.14 | 19.43 | *7* | 17.82 | 15.45 | 13.1 | 10.97 | 9.08 | *-50* | *-53* |
| Land occupation (m²/d) | 9.79 | 11.56 | *18* | 10.26 | 9.2 | 7.33 | 4.72 | 3.28 | *-66* | *-72* |
| 100% organic production |  |  |  |  |  |  |  |  |  |  |
| GHGe (kgCO2eq/d) | 4.09 | 4.68 | *14* | 4.08 | 3.46 | 2.6 | 1.59 | 0.93 | *-77* | *-80* |
| Energy demand (MJ/d) | 16.63 | 18.74 | *13* | 17.15 | 14.92 | 13.06 | 11.17 | 9.26 | *-44* | *-51* |
| Land occupation (m²/d) | 13.35 | 15.74 | *18* | 13.96 | 12.17 | 9.51 | 6.23 | 4.35 | *-67* | *-72* |

Abbreviations: GHGe, greenhouse gas emissions; Obs, observed diet

^1^Values are estimates for incremental 10% increases in the % of protein intake from plant-based foods. The basal scenario (30%) corresponds to the modelled diet when the proportion of protein intake from plant-based foods is set at the observed value of proportion of protein intake from plant-based foods under nutritional, fish consumption limitation and coproducts constraints. Next scenarios increase plant-based foods protein from 30% up to 80%.

**Supplemental Figure 1: Composition (g/d) of the observed and optimized scenarios modelling modelled diets with gradual increase in the proportion of protein intake from plant-based foods^1,2^**


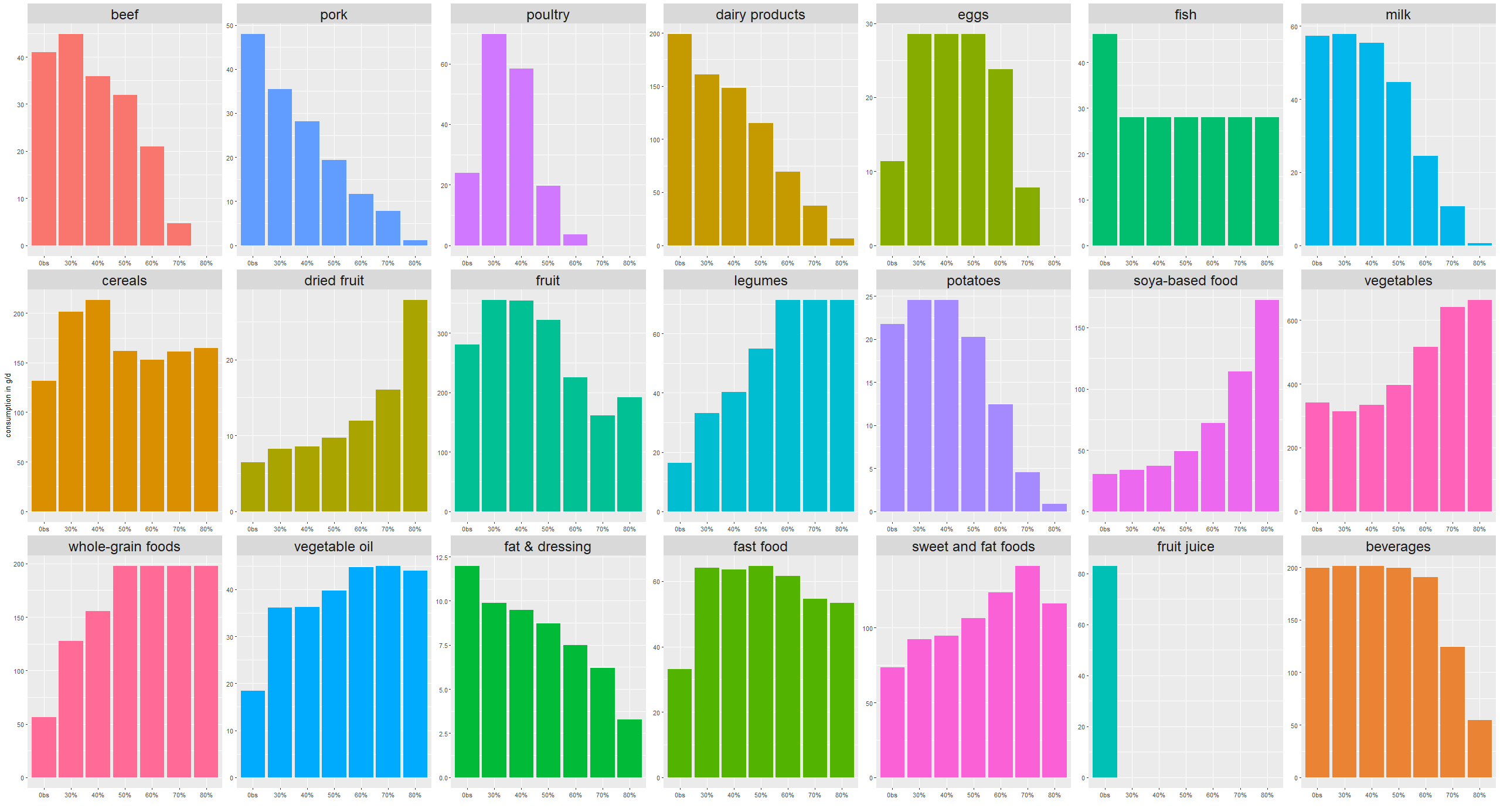
 Abbreviations: Obs, observed diet. ^1^Food group consumption (g/d) in the observed diets and in the modelled diets being nutritionally, culturally and environmentally optimized so as to ensure gradual increase in the proportion of protein intake from plant-based foods. The basal scenario (30%) correspond to the modelled diet when the proportion of protein intake from plant-based foods is set at the observed value of proportion of protein intake from plant-based foods under nutritional, fish consumption limitation and coproducts constraints. Next scenarios increase plant-based foods proteins from 30% up to 80%.

^2^Vegetables include all vegetables and soups, fruit include fresh fruit, fruit in syrup and compote, dried fruit and seeds, fish include seafood, dairy product include yogurts, fresh cheese and cheese, potatoes include other tubers, cereals include breakfast cereal low in sugar, bread semolina, rice and pasta, sweet and fat foods include croissants, pastries, chocolate, biscuits, milky dessert, ice cream, honey and marmalade, cakes, chips, salted oilseeds, salted biscuits, beverages include fruit nectar, syrup, soda (with or without sugar), plant-based beverages (except soy-based), milk consumed with tea/coffee, fast-food include sandwich, prepared foods such as pizza, hamburger, ravioli, panini, salted pancake, etc., soy-based food include tofu, soy-based meat substitute and vegetable patties, soy-based yogurt, soy-based milk, and fat and dressing include sour cream and butter and all fat-based sauces.

**Supplemental Figure 2: Components and scoring of the cDQI**


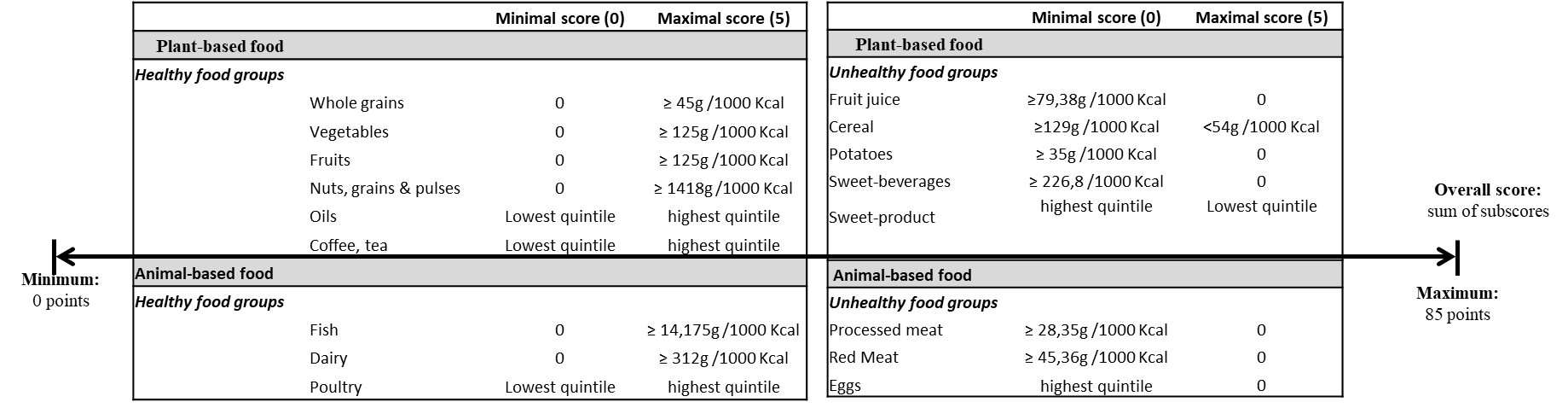


Abbreviations: aDQI, animal diet quality index; cDQI, diet quality index; pDQI, plant diet quality index

**Supplemental Figure 3: Component and scoring of the sPNNS-GS2**


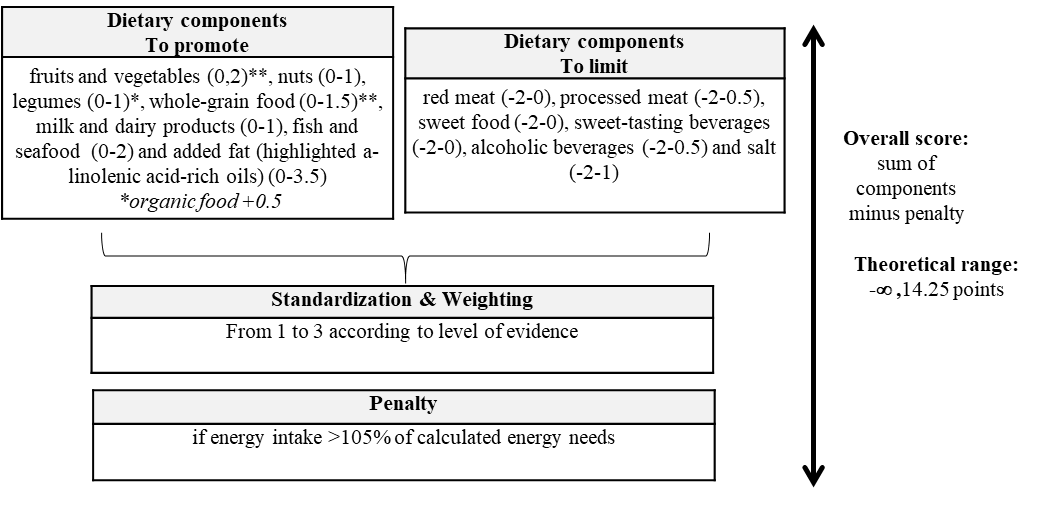


**Supplemental Figure 4: Flowchart of the study**

29,413 participants with data on place of purchase

37,685 participants completed the Org-FFQ

37,305 participants had no missing covariates

34,453 participants were not living overseas

35,196 participants were not under/over-reporters
